# A Case Report of Secondary Postpartum Hemorrhage in a Pregnant Woman With a Mechanical Mitral Valve: Challenges of Anticoagulation

**DOI:** 10.7759/cureus.43778

**Published:** 2023-08-19

**Authors:** Aditi Singh Thakur, Surekha Tayade, Nidhi Makhija, Shikha Toshniwal

**Affiliations:** 1 Department of Obstetrics and Gynaecology, Jawaharlal Nehru Medical College, Datta Meghe Institute of Higher Education & Research, Wardha, IND

**Keywords:** post partum haemorrhage, hemorrhage with prosthetic heart valve, mechanical heart valve in pregnancy, secondary pph in prosthetic valve, pregnancy with prosthetic heart valve

## Abstract

A pregnant woman with rheumatic heart disease always runs the risk of developing both thromboembolic and hemorrhagic symptoms, necessitating careful monitoring of her anticoagulation treatments both throughout pregnancy and after delivery. Postpartum haemorrhage, a hemorrhagic manifestation, can be challenging to control and presents a significant challenge when it comes to beginning anticoagulation after delivery. Thus, pregnancy in these patients is an extremely risky endeavour. Given that these women take anticoagulants, managing these women with artificial heart valves throughout pregnancy can be difficult. The diminished clotting ability in these women may be the cause of postpartum haemorrhage, and a multidisciplinary approach is necessary for a successful treatment. To manage this potentially fatal illness, a well-equipped institution with proper support systems is essential. We present a 23-year-old primigravida who was 39 weeks and three days pregnant and had a repaired aortic valve as well as a prosthetic mitral valve. She was taking warfarin to prevent clotting when she was pregnant.

## Introduction

Any considerable vaginal bleeding between 24 hours after placental delivery and during the course of the next six weeks is referred to as secondary postpartum haemorrhage (SPPH) [1]. Retention of the placenta, endometritis, and delayed placental bed involution are common causes of SPPH. Congenital coagulopathies, cervical malignancy, submucous fibroids, placenta adherence, uterine pseudoaneurysms, and anticoagulant medicines are some less frequent aetiologies [2]. The goal of the initial care is to stabilise the hemodynamics; later, the cause of the bleeding will determine the exact direction of management. If residual placental tissue is suspected, the typical treatment option is uterine evacuation and containment of infection. For continuous bleeding, uterine perforation or uterine pseudoaneurysm, hysterectomy, or arterial embolization may be indicated in certain situations. A pregnant woman with rheumatic heart disease has an increased risk of developing both thromboembolic and hemorrhagic symptoms, necessitating careful monitoring of her anticoagulation therapy during pregnancy and after birth [2].

After birth, initiating anticoagulation following hemorrhagic presentation in the form of postpartum haemorrhage can be quite challenging. Excessive bleeding can cause mortality and morbidity in these women. Due to advancements in the early diagnosis of rheumatic heart disease (RHD) and available facilities for valve replacement, there are now more cases of rheumatic heart disease with pregnancy reporting to tertiary care centres. When a prospective mother receives an anticoagulant, it can be difficult to control the bleeding episode. The risk of thrombosis, miscarriage, early birth, and comorbidities, including valve thrombosis, has been demonstrated to be higher in patients with artificial heart valves who need anticoagulant therapy. The risk of maternal death in individuals can reach up to 3-4%; thus, choosing the right anticoagulant for the gestational age and minimising complications are important considerations [3]. In the second trimester, warfarin is recommended as a dependable, safe, and effective anticoagulant for women with artificial heart valves [3]. However, heparin is suggested during the first trimester to circumvent the danger of embryopathy associated with warfarin. After the first trimester, warfarin must be resumed and withheld just before childbirth to reduce the risk of severe bleeding during delivery [2]. Any haemorrhage during this period should be taken seriously and handled immediately. In this case study, secondary postpartum haemorrhage concerns a pregnant woman on warfarin anticoagulation who had a mitral valve replacement (MVR) and underwent an elective caesarean section.

## Case presentation

**Figure 1:**
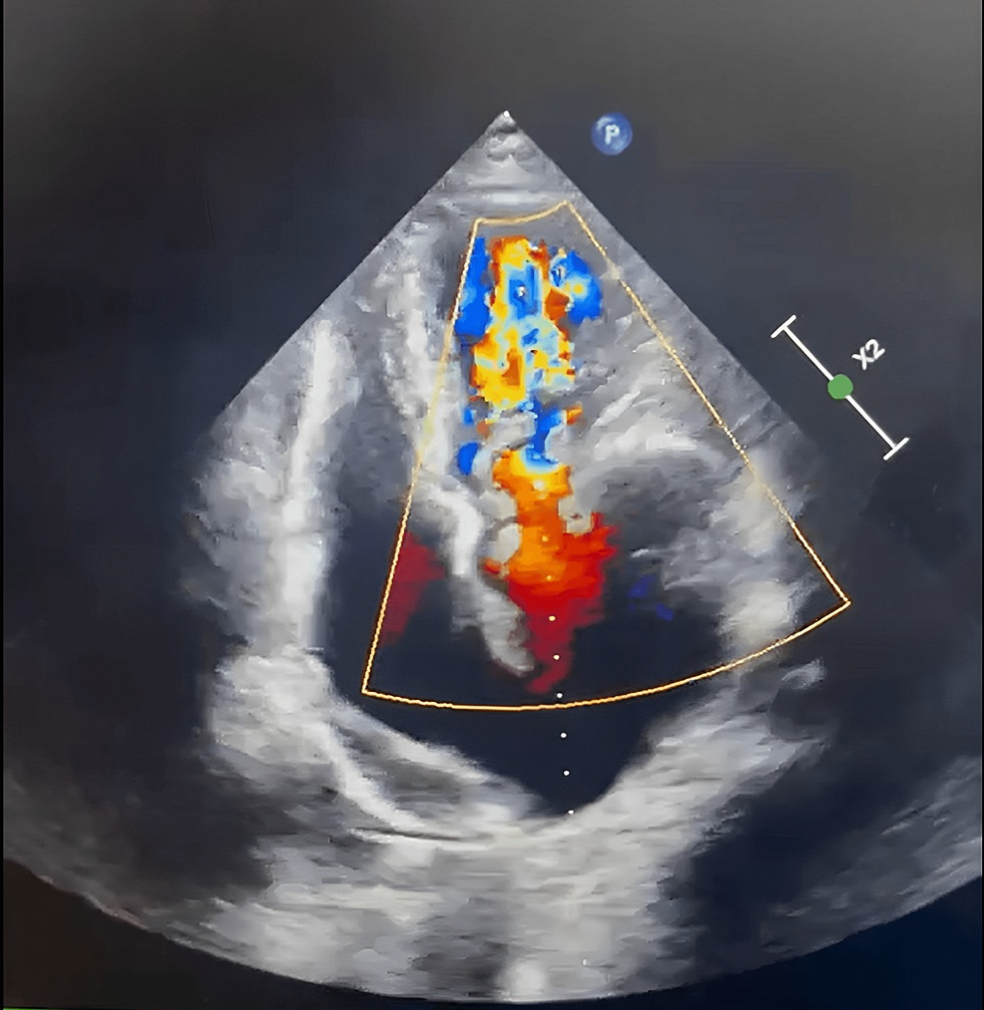
Echocardiography showing mitral stenosis and mitral regurgitation

**Figure 2:**
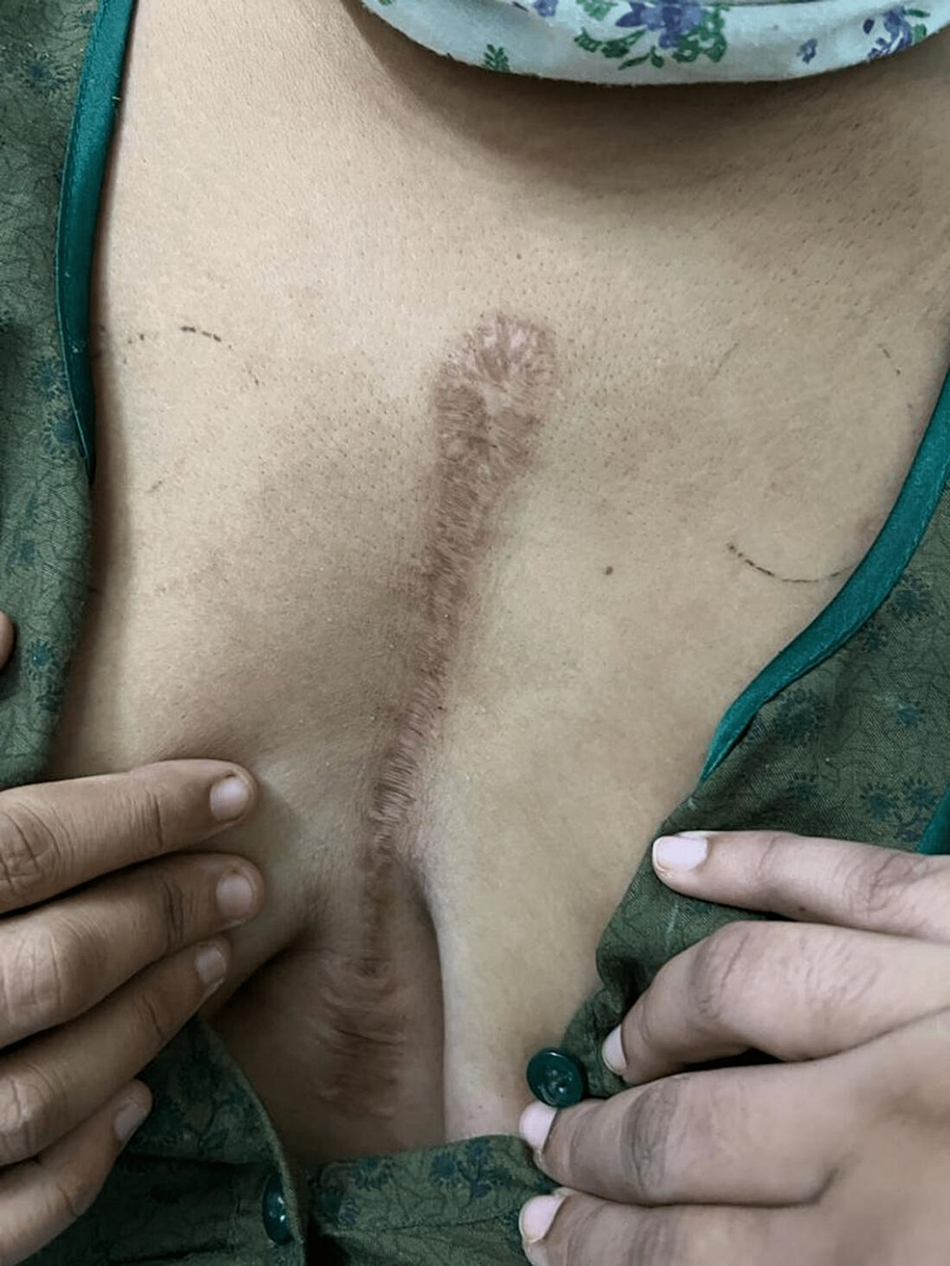
Incision of open heart surgery through which the woman underwent surgery for mitral valve replacement and aortic valve repair

**Figure 3:**
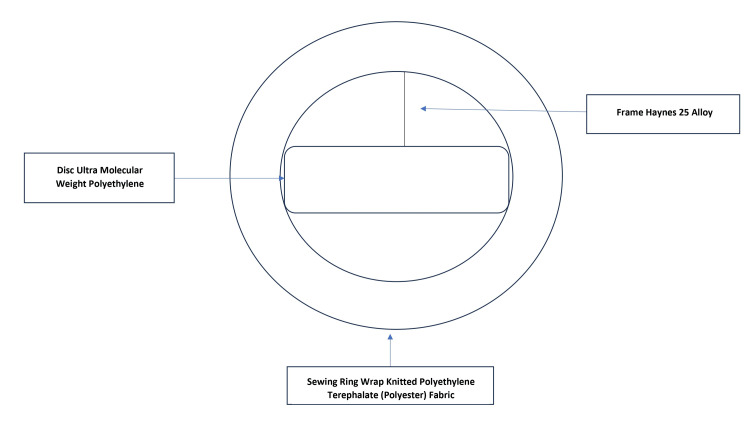
Mechanical prosthetic valve (Chitra™; TTK Healthcare Limited, Chennai, India)

A 23-year-old woman who was 39.3 weeks pregnant presented herself to the outpatient department during emergency hours. She was a naturally occurring case of rheumatic heart disease with an artificial mitral valve replacement. She reported experiencing brief pain in her legs, hip, and wrist joint. Nine years ago, the cause was determined to be rheumatic heart disease after examination. Moderate mitral stenosis, severe mitral regurgitation, moderate aortic regurgitation, and mild pulmonary artery hypertension were all detected by echocardiography (Figure 1). Surgery was performed on her, resulting in removing the anterior mitral leaflet and the chordal preservation of the posterior mitral leaflet (Figure 2). A Chitra™ (TTK Healthcare Limited, Chennai, India) mechanical prosthesis was utilised during the mitral valve replacement (Figure 3). Moreover, the aortic valve was fixed. Since then, she has taken a warfarin 2 mg tablet once daily to prevent clotting.

Her medication was changed to injectable low molecular weight heparin (LMWH) 5000 IU twice daily once it was confirmed that she was pregnant. Weekly partial thromboplastin time (PTT) and prothrombin time (PT) measurements were used for monitoring, and the dose was gradually increased until the third month of pregnancy. Heparin was then stopped, and warfarin was begun instead, taking 1-2 mg orally once daily on alternate days.

When she revealed that she was fully pregnant, a cardiologist's opinion was sought before ending her warfarin tablet medication and starting her on an injectable. An IV infusion of 5000 IU of unfractionated heparin was advised every eight hours. Six hours prior to delivery, she was instructed to discontinue injecting 5000 IU of unfractionated heparin.

The patient was at ease during the examination. She had a vertical scar in the mediastinum from the xiphisternum to 7 cm below the suprasternal notch. Also, in this case, because of the increase in flow across the pulmonic valve, auscultation revealed a systolic murmur best heard in the pulmonary area strongly suggestive of pulmonary hypertension. An examination by an obstetrician found the term relaxed uterus. Upon admission, the patient recommended a 2D echocardiogram, revealing an in situ standard prosthetic valve with no paravalvular leak. Mild tricuspid and aortic regurgitation were present, the biventricular systolic function was normal, and there was no clot formation. A single intrauterine living foetus with an average gestational age of 37.3 weeks was detected by obstetric ultrasound. The coagulation profile was in the expected range.

The mother could not carry the child because of her short stature and the baby's size in her pelvis. The decision was made to perform an elective caesarean section. A cardiologist says heparin should be stopped six hours before a caesarean section. Warfarin was to be continued at 2 mg once a day after 12 hours following surgery, overlapping with heparin post-delivery after ensuring all hemostasis had been achieved. Maintaining PTT and international normalised ratio (INR) was also recommended at 1.5 to 2 times the control value. Heparin was instructed to be stopped after the therapeutic INR was reached. Infective endocarditis prophylaxis and the reserve of blood components (fresh frozen plasma, platelets, and packed red blood cells) were administered.

An elective caesarean section was performed on the patient, who afterwards gave birth to a 2.9-kilogram live baby boy. In the postnatal era, activated PTT was routinely observed. Unfortunately, the woman was discovered to have severe vaginal bleeding with the passage of clots after 24 hours following the caesarean section. Her clothes and two pads are all wet. To exclude retained products, ultrasonography was performed. At that time, the INR was 1.8. To achieve uterine tone, oxytocin was started, and two units of fresh frozen plasma were administered to neutralise the effects of anticoagulants. There were 9.5 g of haemoglobin. To treat any endometritis, an injectable ceftriaxone-sulbactam combination was started. By using these techniques, bleeding was controlled. Anticoagulation wasn't started for another two days. The woman was attentively watched, and her activated partial thromboplastin time (APTT), PT, and INR levels were tracked. When the INR plummeted to 1.1 after being discontinued for three days, LMWH was reintroduced, and eventually, warfarin alone was administered to the patient upon release following bridging therapy.

## Discussion

Because of advancements in the management of valvular heart disease, which necessitates the prescription of anticoagulation during gestation, women of reproductive age with mechanical heart valves (MHV) prefer to become pregnant more frequently [1]. Although only 58% of patients with metallic prostheses experience trouble-free pregnancy and the postpartum period, the anticoagulant medication for MVH counteracts the prothrombotic environment of normal pregnancy [4,5], increasing the risk of thromboembolic and hemorrhagic complications by approximately 7 to 23% in patients with prosthetic heart valves. Anticoagulation has been recommended using a variety of techniques [6,7]. To prevent the teratogenic effects of warfarin, the American Heart Association advises that unfractionated heparin (UFH) or LMWH be prescribed throughout the first trimester [8,9]. After then, warfarin can be started and sustained through delivery in the second trimester. To avoid warfarin-induced mild bleeding, women should transition back to UFH or LMWH at term [10,11].

At a late 35+ weeks, our patient came immediately to the emergency room. Even so, we continued to offer bridging therapy using UFH or LMWH. During an elective caesarean, this stopped the initial postpartum haemorrhage. After 24 hours, however, the woman had vaginal bleeding. After ruling out retained products and initiating therapy to preserve uterine tone, fresh frozen plasma (FFP) was administered to counteract residual anticoagulant effects of chronic warfarin usage. Hemorrhagic problems occurred in six out of 14 pregnancies using prenatal warfarin in descriptive retrospective research by Irani et al. [12]. In addition, Wang et al. [13] reported a retrospective record indicating the rate of hemorrhagic complications was 12.8%, with PPH being responsible for most cases (80%). Some researchers have shown that the incidence of PPH is 2.9-6% in women receiving anticoagulant medications [14-15]. Moreover, Vause et al. found that MHV-positive women had higher rates of maternal mortality and morbidity, with a sizable portion of these morbidities coming from hemorrhagic episodes in the postpartum period [4]. Prothrombin complex concentrate (PCC) transfusion is the most successful treatment for warfarin-induced coagulopathy. It has a number of benefits over FFP, including the potential for more complete restoration and the elimination of volume overload [16-18]. Due to the lack of PCC in our facility, FFP was given to our patient while she was receiving treatment for PPH and subsequently in the intensive care unit (ICU).

Clinical trials to determine when to begin postpartum anticoagulation in pregnant women are insufficient [19,20]. Prophylactic anticoagulation can be begun four to eight hours after a vaginal delivery and eight to 12 hours after a caesarean delivery if there is no sign of bleeding. It is still unknown when it is ideal for women with MHV to continue anticoagulant therapy in the postpartum period because these recommendations are meant for women treated for thromboembolism in pregnancy rather than specifically for women with MHV. Early anticoagulant treatment for our patient wasn't started until two days after the caesarean surgery. After being suspended for three days, LMWH was finally restarted while secondary PPH was treated. After bridging therapy, the patient was eventually released on warfarin alone. It is extremely difficult for clinicians to balance thrombosis and bleeding risks in MHV pregnant women taking anticoagulants [20]. The risk of bleeding increases with a more intensive peripartum anticoagulation regimen, such as one that is therapeutic rather than preventive.

The lack of evidence-based advice for controlling peripartum anticoagulation makes this situation even more challenging while balancing the risk of thrombosis. Taking a multidisciplinary strategy and evaluating clinical and biochemical data closely is advised.

## Conclusions

Ideally, pregnant women with mechanical prosthetic heart valves should be advised about potential pregnancy difficulties (preferably before conception). The mother and baby are at risk when a mechanical prosthetic heart valve is in place. In women with mechanical prosthetic valves, there is a risk of thrombosis, bleeding episodes, and anticoagulation needs to be balanced well. Transfusions of fresh frozen plasma or PCC are effective treatments for warfarin-induced coagulopathy in postpartum haemorrhage. A specialised approach with a multidisciplinary team is necessary for high-risk patients to manage these women throughout pregnancy. The immediate postpartum phase must be addressed with special attention for a healthy outcome.
